# Antimicrobial Peptide Octominin-Encapsulated Chitosan Nanoparticles Enhanced Antifungal and Antibacterial Activities

**DOI:** 10.3390/ijms232415882

**Published:** 2022-12-14

**Authors:** E. H. T. Thulshan Jayathilaka, Chamilani Nikapitiya, Mahanama De Zoysa, Ilson Whang

**Affiliations:** 1 College of Veterinary Medicine and Research Institute of Veterinary Medicine, Chungnam National University, Yuseong-gu, Daejeon 34134, Republic of Korea; 2 National Marine Biodiversity Institute of Korea (MABIK), 75, Jangsan-ro 101 beon-gil, Janghang-eup, Seochun-gun 33662, Republic of Korea

**Keywords:** *Acinetobacter baumannii*, biofilm, *Candida albicans*, chitosan, encapsulation, Octominin, Octominin-CNPs, *Octopus minor*

## Abstract

Antimicrobial peptides (AMPs) have become a key solution for controlling multi-drug-resistant (MDR) pathogens, and the nanoencapsulation of AMPs has been used as a strategy to overcome challenges, such as poor stability, adverse interactions, and toxicity. In previous studies, we have shown the potent antimicrobial activity of Octominin against *Candida albicans* and *Acinetobacter baumannii.* This study is focused on the nanoencapsulation of Octominin with chitosan (CS) and carboxymethyl chitosan (CMC) as a drug delivery system using the ionotropic gelation technique. Octominin-encapsulated CS nanoparticles (Octominin-CNPs) had an average diameter and zeta potential of 372.80 ± 2.31 nm and +51.23 ± 0.38 mV, respectively, while encapsulation efficiency and loading capacity were 96.49 and 40.20%, respectively. Furthermore, Octominin-CNPs showed an initial rapid and later sustained biphasic release profile, and up to 88.26 ± 3.26% of the total Octominin release until 96 h. Transmission electron microscopy data showed the irregular shape of the Octominin-CNPs with aggregations. In vitro and in vivo toxicity of Octominin-CNPs was significantly lower than the Octominin at higher concentrations. The antifungal and antibacterial activities of Octominin-CNPs were slightly higher than those of Octominin in both the time-kill kinetic and microbial viability assays against *C. albicans* and *A. baumannii*, respectively. Mode of action assessments of Octominin-CNPs revealed that morphological alterations, cell membrane permeability alterations, and reactive oxygen species generation were slightly higher than those of Octominin at the tested concentrations against both *C. albicans* and *A. baumannii*. In antibiofilm activity assays, Octominin-CNPs showed slightly higher biofilm inhibition and biofilm eradication activities compared to that of Octominin. In conclusion, Octominin was successfully encapsulated into CS, and Octominin-CNPs showed lower toxicity and greater antimicrobial activity against *C. albicans* and *A. baumannii* compared to Octominin.

## 1. Introduction

Commercially available antibiotics are becoming less effective due to the rapid development of antibiotic resistance [1]. Therefore, scientists are focusing on alternatives to antibiotics. Antimicrobial peptides (AMPs) are a group of host defense molecules and have been shown to be reliable in controlling a wide range of pathogenic bacteria, fungi, and viruses [2,3]. AMPs comprise a short sequence of amino acids with cationic charge, higher hydrophobicity, and amphipathic nature [4]. These features facilitate a broad spectrum of antimicrobial activities of AMPs, such as induction of morphological changes, alteration of membrane permeability, generation of reactive oxygen species (ROS), DNA damage, and inhibition of protein synthesis [5]. Despite their potential antimicrobial activity, clinical usage of AMPs has been limited owing to the poor in vivo stability, adverse interactions with the host immune system, and possible toxicity development [6,7]. Cyclization, terminal modification, and unusual amino acid introduction are common modifications that can be introduced to AMPs to overcome these limitations before therapeutic applications [8]. The encapsulation of AMPs into nanoparticles (NPs) has grown into a major solution to improve stability by safeguarding proteolytic degradation, targeted drug delivery of AMPs to the site of infection, and lowering toxicity with pharmacokinetics modifications [9,10].

With the rapid development of nanotechnology, its applications have cooperated in biomedical and microbiological fields for advanced therapeutic applications [11]. Drug encapsulation into NPs has shown potential for drug delivery and efficient and synergistic drug action at the targeted site [12,13]. Chitosan (CS), a linear polysaccharide composed of randomly distributed β-linked D-glucosamine and N-acetyl-D-glucosamine units, has been widely used for nanoencapsulation [14]. CS has been confirmed to be biocompatible, biodegradable, and non-toxic as an excipient in drug formulation [15]. Furthermore, as a biomaterial, CS has a track record of its inherent multiple antimicrobial modes of action against a broad spectrum of organisms, including multidrug-resistant (MDR) microorganisms [16]. Consequently, AMPs encapsulation into chitosan NPs (CNPs) is a promising solution to overcome the therapeutic limitations of AMPs [17]. Several studies have been conducted on AMPs encapsulation using CNPs. For instance, Zhu et al. demonstrated AMP encapsulation using CS quaternary ammonium salt-induced antibacterial activity against *Escherichia coli* [18].

Octominin is a novel synthetic AMP based on the defense protein of *Octopus minor*, which consists of 23 amino acids, a total net charge of +5, a hydrophobic ratio of 43%, and 1.86 kcal/mol on the Boman index [19]. Our previous studies reported the anticandidal and antibacterial activities of Octominin against *Candida albicans* [20] and *Acinetobacter baumannii* [21], respectively. In this study, we mainly focused on encapsulating Octominin into the core–shell structure of CNPs to enhance the antimicrobial activity against *C. albicans* and *A. baumannii*. We evaluated the characteristics of Octominin encapsulated CNPs (Octominin-CNPs) based on their size, zeta potential, morphology, encapsulation efficiency (EE), loading capacity (LC), and AMP release profiles. We then confirmed its antimicrobial activity against *C. albicans* and *A. baumannii* using a time-kill kinetic assay and viability test. Furthermore, morphological changes, membrane permeability alterations, ROS generation, and antibiofilm activity were determined to confirm the efficiency and the mode of action of Octominin-CNPs compared to unencapsulated Octominin.

## 2. Results

### 2.1. Optimization of the Encapsulation of Octominin-CNPs

To determine the optimum ratio of Octominin:CMC:CS for encapsulation reaction, we used different ratios of Octominin while keeping the other two components (CMC and CS) at a constant ratio. Reaction mixture 4 (CS:CMC:Octominin-0.4:2:1) showed the highest EE (96.49%) (Table 1). Even though reaction mixture 5 (CS:CMC:Octominin-0.4:2:1.5) showed a higher LC (48.85%) than reaction mixture 4 (40.20%), it had a lower EE (78.16%). Considering the EE and average particle size, a CS:CMC:Octominin ratio of 0.4:2:1 was selected as the optimum ratio for further encapsulation experiments.

### 2.2. Preparation, Characterization, and Release of Octominin-CNPs

Octominin-CNPs were obtained using the ionotropic gelation method with CS:CMC:Octominin at the ratio of 0.4:2:1. The Octominin-CNPs suspension appeared opalescent with no particle aggregation. Initially, macroscopically appreciable aggregates were observed in the suspension, and with slight sonication, the Octominin-CNPs were fully dispersed without visible aggregates. The laser diffraction particle size analysis of Octominin-CNPs revealed that the diameter is slightly higher than CNPs; the obtained particle diameters were 372.8 ± 2.3 and 246.81 ± 1.98 nm, for Octominin-CNPs and CNPs, respectively. The cationic nature of CS produced positive zeta potentials of +51.23 ± 0.38 and +59.33 ± 3.63 mV for Octominin-CNPs and CNPs, respectively, in PBS at pH 7.4.

**Figure 1:**
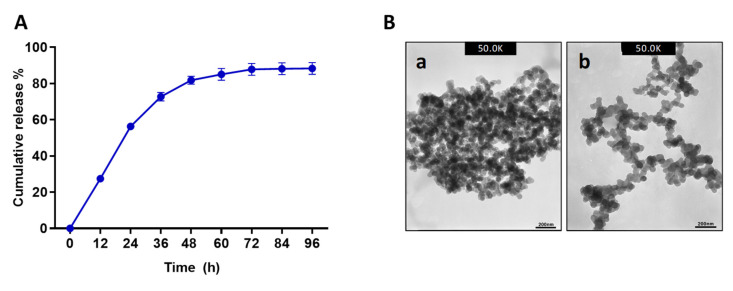
The cumulative release profile of Octominin-CNPs and morphology of Octominin-CNPs and CNPs (**A**) The cumulative release profile of Octominin from Octominin-CNPs in PBS (pH 7.4) at 37 °C (mean ± SD, n = 3). (**B**) Morphological observation of Octominin-CNPs and CNPs under transmission electron microscopy (TEM) analysis. TEM micrographs of (**a**) CNPs (**b**) Octominin-CNPs.

The EE and LC were measured by quantifying the remaining Octominin in the supernatant after the isolation of NPs. Octominin-CNPs had an EE of 96.49% and an LC of 40.20%. Peptide release kinetics of Octominin-CNPs were observed in PBS at pH 7.4 for 96 h (Figure 1A). The peptide release profile showed a biphasic release pattern with an initial rapid linear release of up to 56.2% until 24 h, and a later sustained release rate to reach the maximum cumulative release of 88.26% at 96 h. Transmission electron microscopy (TEM) analysis was conducted to observe the morphology of the Octominin-CNPs versus CNPs (Figure 1B). The TEM micrographs of both NPs showed round-shaped particles with excessive aggregation. The aggregation of Octominin-CNPs was relatively low compared to that of CNPs.

### 2.3. Cytocompatibility and In Vivo Toxicity of Octominin-CNPs vs. Octominin

**Figure 2:**
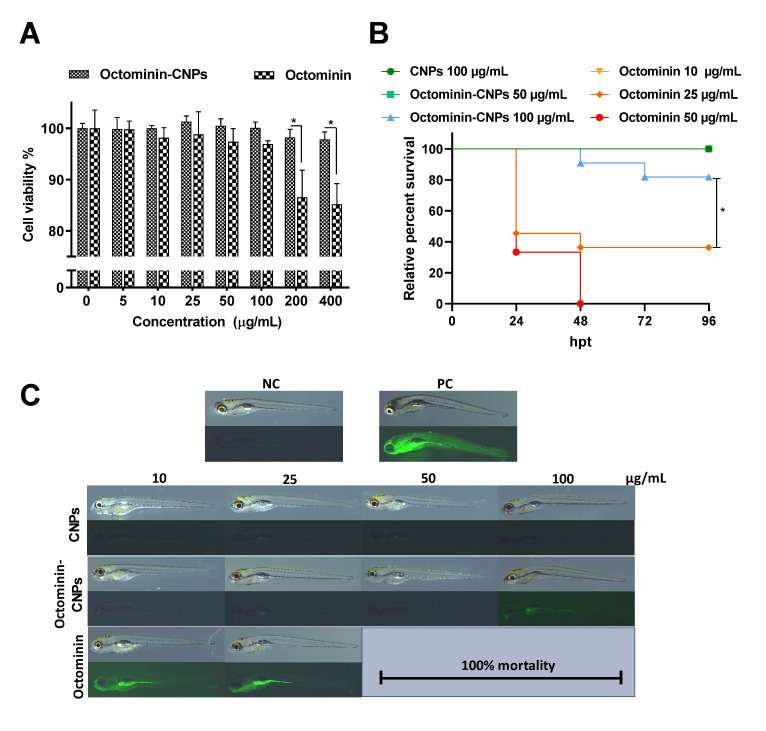
In vitro and in vivo toxicity determination of the Octominin-CNPs vs. Octominin. (**A**) 3-(4,5-dimethylthiazol-2-yl)-2,5-diphenyltetrazolium bromide (MTT) assay was conducted to determine Octominin-CNPs/Octominin cytotoxicity using human embryonic kidney 293 (HEK 293) cells. Cells (2.0 × 10^5^ cells/mL) were treated with different concentrations of Octominin-CNPs and Octominin (0–400 µg/mL) and incubated at 37 °C for 24 h in a 5% CO_2_ incubator. The supernatant was then replaced with MTT to determine the viability of the cells. * *p* < 0.05. The error bars indicate the mean ± SD (n = 3). (**B**) Relative present survival (RPS) of zebrafish larvae with Octominin-CNPs, Octominin, CNPs treatments. Zebrafish larvae at 60 h post-fertilization (hpf) were treated with different concentrations of Octominin-CNPs and Octominin (0–100 µg/mL). RPS was determined at 12 h intervals. * *p* < 0.05, The error bars indicate the mean ± SD (n = 10). (**C**) Octominin-CNPs, Octominin induced reactive oxygen species (ROS) generation determination in zebrafish larvae. At 96 h post-treatment (hpt), larvae were stained with 2′7′dichlorodihydro-fluorescein diacetate (H_2_DCFDA) and observed for green fluorescence.

The cytotoxicity of Octominin-CNPs and Octominin was evaluated using human embryonic kidney 293 (HEK 293) cells (Figure 2A). Both Octominin-CNPs and Octominin showed a minimal reduction in cellular viability up to 100 µg/mL. However, when the concentration of Octominin increased (200 and 400 µg/mL), a significant (*p* < 0.05) reduction in viability was observed with Octominin treatment compared to that of Octominin-CNPs. At the highest tested concentration (400 µg/mL), Octominin-CNPs exhibited viability of 97.83%, whereas free Octominin had 85.19% viability.

In vivo toxicity determination and ROS generation assays confirmed the non-toxic nature of Octominin-CNPs compared to that of Octominin at the tested concentrations. In particular, Octominin caused 100% mortality at 50 and 100 µg/mL and 40% relative percent survival (RPS) at 25 µg/mL, whereas Octominin-CNPs did not cause any mortality or growth/behavioral abnormalities up to 50 µg/mL (Figure 2B,C). At 100 µg/mL, the Octominin-CNPs had 80% RPS and did not show any abnormalities in the remaining larvae. In the ROS generation assay, Octominin showed a slight ROS signal at 10 µg/mL and a relatively strong signal at 25 µg/mL, while Octominin-CNPs showed a very slight signal for ROS detection only at 100 µg/mL (Figure 2C).

### 2.4. Antifungal and Antibacterial Activities of Octominin-CNPs vs. Octominin

**Figure 3:**
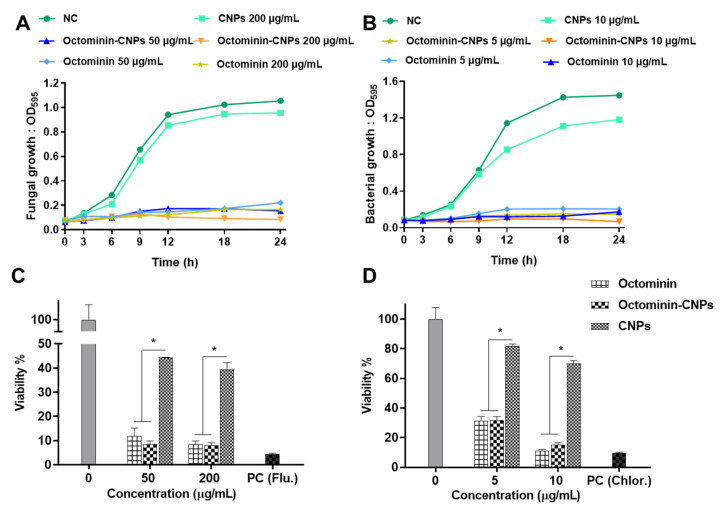
Time-kill kinetics and fungal/bacterial viability analysis with the treatment of Octominin-CNPs, Octominin, and CNPs. Octominin-CNPs, Octominin and CNPs were treated for (**A**) *Candida albicans* at 50 (minimum inhibitory concentration; MIC) and 200 µg/mL (minimum fungicidal concentration; MFC) and (**B**) *Acinetobacter baumannii* at 5 (MIC) and 10 µg/mL (minimum bactericidal concentration; MBC). Fungal/bacterial growth was measured with an optical density at 595 nm (OD_595_) at 0, 3, 6, 9, 12, 18, and 24 h and derived time-kill kinetic graphs. Viability assay of (**C**) *C. albicans* and (**D**) *A. baumannii* with treatments of Octominin-CNPs, Octominin, and CNPs. Fungi/bacteria were treated with MIC and MFC/MBC levels of Octominin-CNPs or Octominin or CNPs, and viability was measured using an MTT assay after 24 h of treatment. * *p* < 0.05, The error bars indicate the mean ± SD (n = 3). OD_595_; optical density at 595 nm, NC—negative control, PC—positive control, Flu—Fluconazole, Chlor—Chloramphenicol.

In previous studies, Octominin was identified to have antifungal activity against *C. albicans* with minimum inhibitory concentration (MIC) and minimum fungicidal concentration (MFC) of 50 and 200 µg/mL [20], and antibacterial activity against *A. baumannii* with MIC and minimum bactericidal concentrations (MBC) of 5 and 10 µg/mL, respectively [21]. The antimicrobial activities of Octominin-CNPs and Octominin were compared against those of *C. albicans* and *A. baumannii* at peptide concentrations of MICs and MBC/MFC. Figure 3 depicts the time-kill kinetics activity and fungal/bacterial viability reduction with each Octominin-CNPs and Octominin treatment. In time-kill kinetic assays, CNPs treated samples grew slightly below the negative controls, showing minor antifungal and antibacterial activities against *C. albicans* and *A. baumannii*, respectively. In the early hours, Octominin had higher antifungal and antibacterial activities than Octominin-CNPs. However, in the late hours (6 h post treatment; hpt), Octominin-CNPs had slightly higher antifungal and antibacterial activities than the respective concentrations of free Octominin.

Viability assessment was conducted on Octominin-CNPs and Octominin-treated *C. albicans* and *A. baumannii* using the 3-(4,5-dimethylthiazol-2-yl)-2,5-diphenyltetrazolium bromide (MTT) assay. At MIC levels, the reduction in *C. albicans* viability was higher in Octominin-CNPs (8%) than in Octominin (11%), but in *A. baumannii*, both treatments showed the same viability reduction (30%). The *C. albicans* group showed similar fungicidal effects at the MFC level (8%) in both treatments. However, at the MBC level, *A. baumannii* group had slightly lower bacterial viability in the Octominin treatment (11%) group than in the Octominin-CNPs treatment (15%).

### 2.5. Morphological Alteration of C. albicans and A. baumannii with Octominin-CNPs vs. Octominin

**Figure 4:**
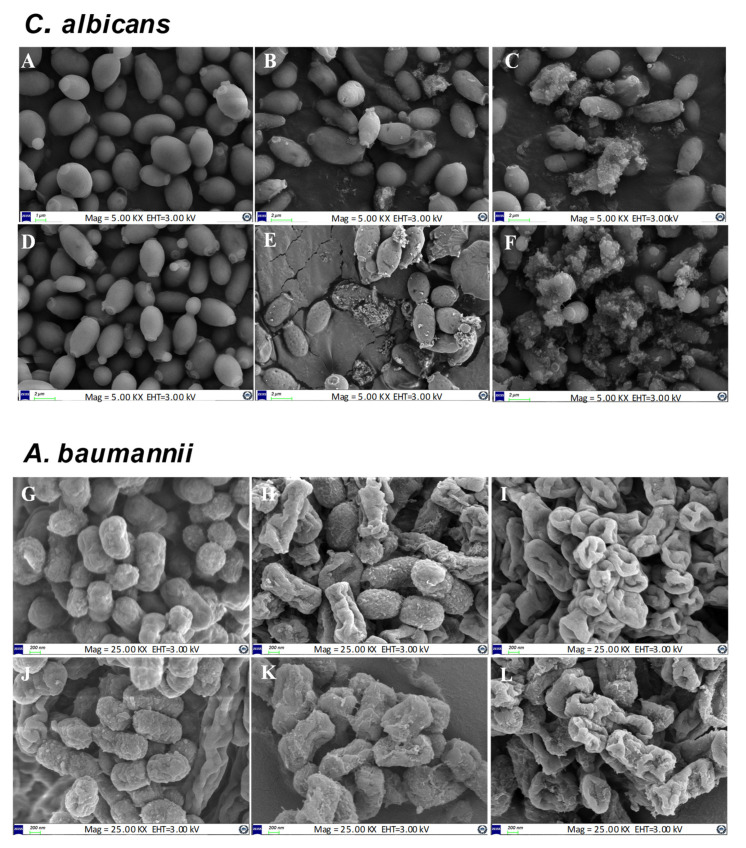
Comparison of morphological and structural alterations of *C. albicans* and *A. baumannii* upon Octominin-CNPs, Octominin, and CNPs treatments. *C. albicans* was treated with (**A**) 1 × phosphate buffered saline (PBS), (**B**) Octominin MIC (50 µg/mL), (**C**) Octominin MFC (200 µg/mL), (**D**) CNPs (200 µg/mL), (**E**) Octominin-CNPs (50 µg/mL), and (**F**) Octominin-CNPs (200 µg/mL). *A. baumannii* was treated with (**G**) PBS, (**H**) Octominin MIC (5 µg/mL), (**I**) Octominin MBC (10 µg/mL), (**J**) CNPs (10 µg/mL), (**K**) Octominin-CNPs (5 µg/mL), and (**L**) Octominin-CNPs (10 µg/mL). After 12 h of treatment, fungi/bacteria were fixed with glutaraldehyde (5%) and coated with platinum. Fungi/bacteria were observed under field emission scanning microscopy. The scale bar for *C. albicans* is 2 µm and for *A. baumannii* is 200 nm.

Field emission electron microscopy (FE-SEM) analysis was conducted on *C. albicans* and *A. baumannii* to determine the intensity of the morphological alterations after treatment with Octominin-CNPs, Octominin, and CNPs (Figure 4). In *C. albicans*, CNPs treatment did not cause any changes on the surface of fungal cells and had the same morphological structure as that of the negative control. A comparison of the antifungal effects of Octominin-CNPs and Octominin on *C. albicans* showed that encapsulated peptides had superior activity by causing greater damage to the fungal surface. Specifically, the MIC (50 µg/mL) level of Octominin caused minor damage with small pore formation, whereas Octominin-CNPs caused significant damage with substantial pore formation and cell shrinkage. At MFC (200 µg/mL), Octominin caused significantly high damage to the fungal surface with cell shrinkage and cell disruption in a few cells, while Octominin-CNPs caused total cell disruption, causing the highest damage to fungal cells. A similar pattern of morphological alterations was observed in the *A. baumannii* samples. However, CNPs caused minor alterations in bacterial cells with surface shrinkage compared with the negative control. Octominin-CNPs produced superior activity against *A. baumannii* at the MIC (5 µg/mL) and MBC (10 µg/mL) levels compared to each concentration of Octominin. Specifically, the number of cells with bacterial cell shrinkage was higher in the Octominin-CNPs treated cells than in the Octominin alone at the MIC level. At the MBC level, both groups showed bacterial cell damage with a hole formation; however, the severity was higher in the Octominin-CNPs-treated group.

### 2.6. Membrane Permeability Alteration with Treatments of Octominin-CNPs vs. Octominin

**Figure 5:**
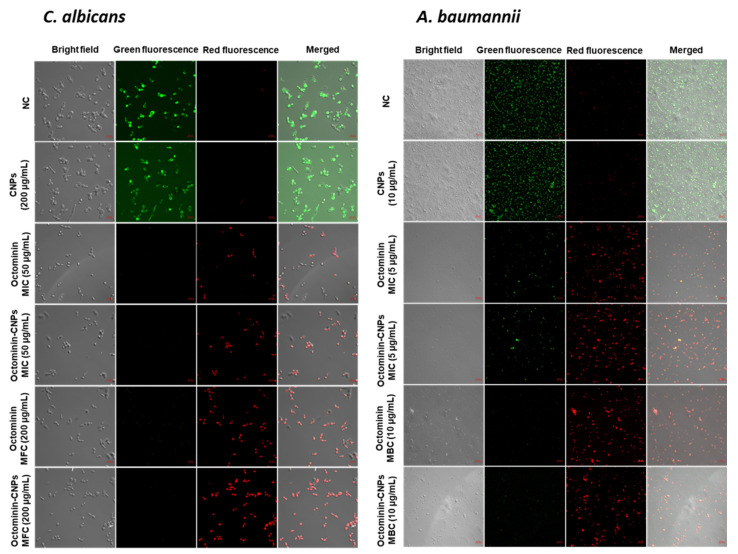
Comparison of membrane permeability alteration in *C. albicans* and *A. baumannii* with treatments of Octominin-CNPs, Octominin, and CNPs. *C. albicans* was treated with PBS (negative control), CNPs (200 µg/mL), Octominin MIC (50 µg/mL), Octominin MFC (200 µg/mL), Octominin-CNPs MIC (50 µg/mL), Octominin-CNPs MFC (200 µg/mL). *A. baumannii* was treated with PBS (negative control), CNPs (10 µg/mL), Octominin MIC (5 µg/mL), Octominin MBC (10 µg/mL), Octominin-CNPs MIC (5 µg/mL), Octominin-CNPs MBC (10 µg/mL). After 12 h of treatment fungi/bacteria were separated and stained with propidium iodide (PI) for membrane permeability altered cell identification and fluorescein diacetate (FDA) for viable cell determination. Fungi/bacteria were observed under confocal microscopy at excitation and emission wavelengths at 535 and 617 nm, respectively for red fluorescence and 488 and 535 nm, respectively for green fluorescence. The scale bar denotes 10 µm. NC—Negative control.

Propidium iodide (PI) uptake coupled with fluorescein diacetate (FDA) staining was conducted in *C. albicans* and *A. baumannii* treated with Octominin-CNPs or Octominin or CNPs. PI can penetrate cells with altered permeability and produce red fluorescence, whereas the FDA produces green fluorescence in viable cells. CNPs treated samples in both groups had only green-fluorescent cells, indicating that membrane permeability did not change upon CNPs (Figure 5). However, when the *C. albicans* and *A. baumannii* samples were treated with MIC levels of Octominin-CNPs or Octominin or CNPs, red fluorescent cells were observed, and green-fluorescent cells were observed at a low level. Treatment with MBC levels of both Octominin-CNPs and free Octominin produced only red fluorescence with the absence of green fluorescence. A significantly high level of red fluorescence was observed in Octominin-CNPs treatment in both MIC and MFC/MBC in *C. albicans* and *A. baumannii*, suggesting that Octominin-CNPs possessed higher potency to induce membrane permeability alterations.

### 2.7. ROS Generation with Octominin-CNPs vs. Octominin

**Figure 6:**
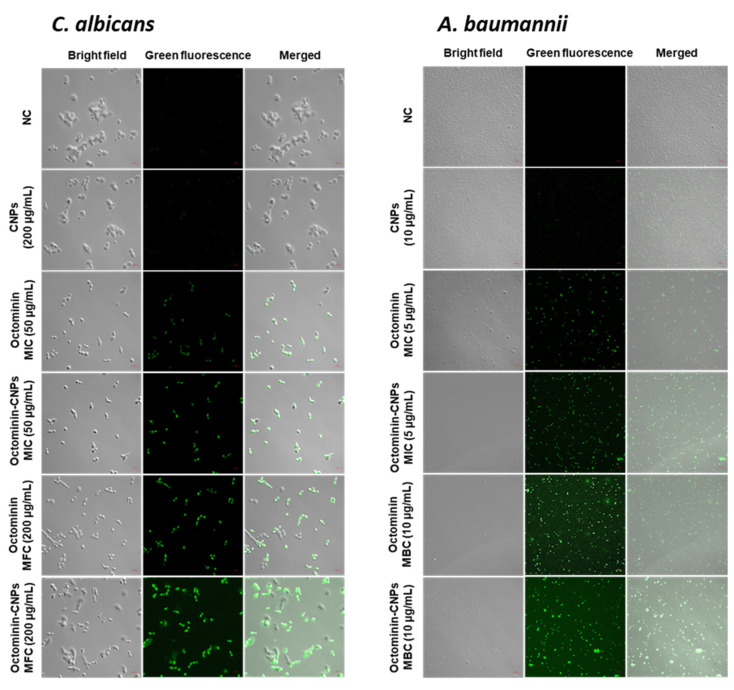
Comparison of ROS generation in *C. albicans* and *A. baumannii* with treatments between Octominin-CNPs, Octominin, and CNPs. *C. albicans* were treated with PBS (negative control), CNPs (200 µg/mL), Octominin MIC (50 µg/mL), Octominin MFC (200 µg/mL), Octominin-CNPs MIC (50 µg/mL), and Octominin-CNPs MFC (200 µg/mL). *A. baumannii* was treated with PBS (negative control), CNPs (10 µg/mL), Octominin MIC (5 µg/mL), Octominin MBC (10 µg/mL), Octominin-CNPs MIC (5 µg/mL), and Octominin-CNPs MBC (10 µg/mL). After 12 h of treatment, fungi/bacteria were separated and stained with H_2_DCFDA. Fungi/bacteria were observed under confocal microscopy at excitation and emission wavelengths of 488 and 535 nm, respectively. The scale bar denotes 10 µm. NC—Negative control.

The H_2_DCFDA staining was conducted to compare ROS generation capacity in Octominin-CNPs, Octominin, and CNPs-treated *C. albicans* and *A. baumannii*. In both groups, CNPs produced no green fluorescence, proving that CNPs do not cause ROS generation by itself in *C. albicans* and *A. baumannii*. In both groups, Octominin-CNPs caused a slightly higher green fluorescence level than Octominin at each MIC and MFC/MBC level (Figure 6). These results suggested that Octominin-CNPs triggered higher ROS generation than Octominin.

### 2.8. Antibiofilm Activity of Octominin-CNPs vs. Octominin

**Figure 7:**
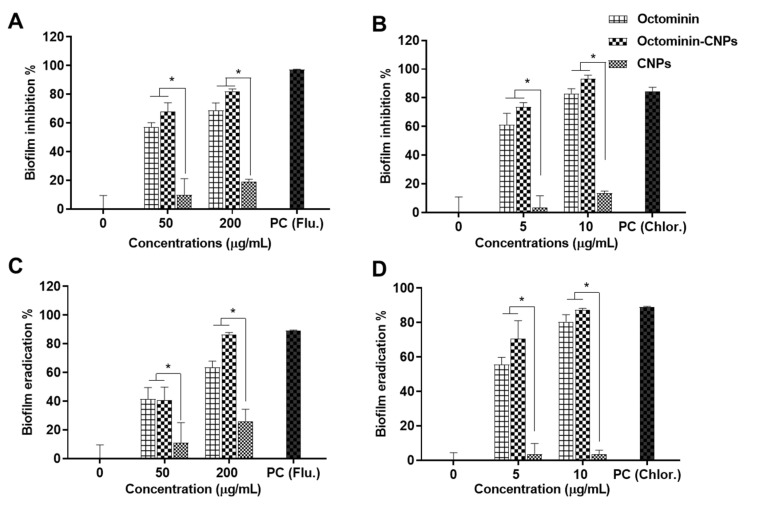
Comparison of antibiofilm activity in *C. albicans* and *A. baumannii* upon Octominin-CNPs, Octominin, and CNPs. Biofilm inhibition of (**A**) *C. albicans* and (**B**) *A. baumannii* with Octominin-CNPs, Octominin, and CNPs treatments. Biofilm eradication assay on pre-formed biofilms of (**C**) *C. albicans* and (**D**) *A. baumannii* upon treatments of Octominin-CNPs, Octominin, and CNPs. Remaining biofilms after incubation were quantified with the crystal violet staining method. * *p* < 0.05: the error bars indicate the mean ± SD (n = 3).

Biofilm formation inhibition and biofilm eradication assays (crystal violet; CV staining) were conducted on *C. albicans* and *A. baumannii* treated with Octominin-CNPs, Octominin, and CNPs (Figure 7). CNPs showed relatively low biofilm inhibition capacities of 9.90 and 19.25% in *C. albicans* at 50 and 200 µg/mL, respectively (Figure 7A), and 3.51 and 13.59% in *A. baumannii* at 5 and 10 µg/mL, respectively (Figure 7B). Octominin-CNPs showed greater biofilm inhibition activity than Octominin at each tested concentration in both *C. albicans* and *A. baumannii*. Octominin-CNPs had *C. albicans* biofilm inhibition levels of 67.97 and 82.28% at MIC and MFC levels, respectively, whereas Octominin as a free drug had 57.12 and 68.91% inhibition, respectively. In *A. baumannii*, Octominin-CNP showed 73.70 and 93.21% biofilm formation inhibition at the MIC and MBC levels, respectively, while Octominin showed 61.30 and 83.06%, respectively. Similar to biofilm inhibition, CNPs showed minor biofilm eradication capabilities of 10.93 and 26.18% for *C. albicans* at 50 and 200 µg/mL, respectively (Figure 7C), and 3.73 and 3.60% for *A. baumannii* at 5 and 10 µg/mL, respectively (Figure 7D). In the *C. albicans* group, Octominin-CNPs had biofilm eradication activities of 40.73 and 86.61% at MIC and MFC levels, respectively, while Octominin had eradication rates of 41.50 and 63.57%, respectively. Octominin-CNPs had greater *A. baumannii* biofilm eradication levels of 70.74 and 87.30% at MIC and MBC, respectively, whereas Octominin had 55.66 and 80.53% eradication, respectively.

## 3. Discussion

To examine the therapeutic effects and targeted drug delivery capacities of CS-based nano-encapsulated AMPs, Octominin was encapsulated into CNPs and its characteristic physiochemical properties, release kinetics, in vitro and in vivo toxicity, antifungal, and antibacterial efficiencies were determined. The repulsion between cationic AMPs and CS is a limiting factor for encapsulation. The incorporation of the anionic compounds into the ionotropic gelation as a crosslinker is an excellent strategy to overcome this repulsion. In this regard, Piras et al. [17] demonstrated the encapsulation of renin substrate I (cationic, and hydrophobic) into CNPs in the presence of sodium tripolyphosphate (TPP) as the anionic crosslinker. In this study, we used CMC, a negatively charged water-soluble polymer, to form a favorable crosslink between Octominin and CS [22]. After the initial 40 min of stirring, the Octominin-CMC mixture displayed microaggregates, and the subsequent introduction of CS facilitated the assembly of dense peptide-rich NPs covering the CS layer.

Previously AMPs named LL37 was encapsulated into poly lactic-co-glycolic acid (PLGA) and synthesized the NPs having a particle diameter of ~300 nm and 70% EE, but it had a lower LC (0.10%) [23]. Moreover, AMPs CM11 was encapsulated into hyaluronic acid-coated CNPs with a particle diameter of 190 nm and had an EE of 60% [24]. Compared to these AMP encapsulation studies, Octominin-CNPs had an average particle diameter of 372.80 ± 2.31 nm and higher EE (96.49%) and LC (40.60%), confirming efficient encapsulation of Octominin with CNPs. In the encapsulation process, Octominin induced the conformational and charge rearrangements in CS, which were reflected in higher particle diameter and lower zeta-potential values in Octominin-CNPs compared to that of CNPs. Similarly, Piras et al. [25] showed that CS-encapsulated temporin B had a higher diameter and lower zeta potential than blank CNPs. Furthermore, TEM analysis of the NPs showed similar particle textures in both samples. However, Octominin-CNPs showed a lower level of aggregation. This effect might also be due to confirmational and zeta-potential deviations in CS after ionotropic gelation with Octominin. The Octominin release profile from the Octominin-CNPs followed a biphasic pattern with an initial burst (up to 24 h) followed by a slow and sustained release. A similar pattern of biphasic release profile was beneficial for the LL37 encapsulated poly (lactic-co-glycolic acid) NPs. It promoted wound healing by an initial rapid release of a high concentration of LL37 at the wound site to activate the therapeutic effect and subsequently sustained release to maintain the LL37 concentration for a prolonged time after the initial administration of the nanoparticle [23]. The biphasic initial rapid and later sustained release pattern of Octominin-CNPs was beneficial for the rapid fungicidal and bactericidal action to eradicate microorganisms, prevent the regrowth of microbes, and develop resistance development against Octominin.

Although the cytotoxicity level of Octominin was very low (up to 100 µg/mL), it reduced cell viability in a concentration-dependent manner in HEK 293 cells [19]. Nevertheless, an in vitro cytocompatibility evaluation of the Octominin-CNPs revealed its excellent capacity to reduce the cytotoxicity of Octominin, even at concentrations above 200 µg/mL. This result confirmed that the encapsulation of Octominin into CNPs may improve the usage capability of Octominin at high doses with minimal toxicity and enhanced therapeutic activities with sustained drug release patterns. Nwokwu et al. [26] demonstrated a similar result in boosting the anticancer effect of gedunin against non-small-cell lung cancer (NCI-H292) cells while reducing its cytotoxicity towards lung fibroblast cells (MRC-5) after encapsulation into CNPs. Previously, Octominin applications were limited in the zebrafish larvae model because of its toxicity above 25 µg/mL [19]. However, in this study, in vivo toxicity determination using zebrafish larvae confirmed the non-toxic nature of Octominin-CNPs up to 50 µg/mL and low ROS generation at high doses (100 µg/mL), which ensures the possibility of Octominin-CNPs application in animal models at higher concentrations as a therapeutic agent.

Apart from being an encapsulating agent, the most investigated property of CS is its antimicrobial effect against a wide range of target organisms, such as algae, bacteria, yeasts, and fungi [16]. 2,6-diamino chitosan (2,6-DAC) is a CS-derived cationic polymer with excellent synergistic antimicrobial effects with various antibiotics against multidrug resistance (MDR) *A. baumannii* and methicillin-resistant *Streptococcus aureus* [27]. Correspondingly, our study observed higher antifungal and antibacterial activities of Octominin-CNPs compared to Octominin owing to the synergistic action of CS and its encapsulated AMP. Even though the time-kill kinetics assay and fungal/bacterial viability assays showed marginally similar antimicrobial activity in Octominin-CNPs compared to Octominin against *C. albicans* and *A. baumannii*, results of mode of action proved the superior antifungal and antibacterial activities of Octominin-CNPs. There are experimental difficulties with prolonged fungi and bacteria incubation time in culture media because nutrient depletion leads to spontaneous microbial death [28]. It is noteworthy that antimicrobial activity assays in this study were limited to 24 h to quantify the activities of Octominin-CNPs against Octominin. Otherwise, we predicted that Octominin-CNPs would have produced prolonged fungal/bacterial growth inhibition against *C. albicans* and *A. baumannii* in time-kill kinetics compared to Octominin.

Our previous studies showed that Octominin possesses multiple modes of action against *C. albicans* and *A. baumannii* [20,21]. However, in this study, we observed an increase in the intensity of these modes of action with Octominin-CNPs treatments compared to Octominin treatment. Although the precise mode of action of CS for antimicrobial activity has not been well discussed, few studies have proposed that the physiochemical properties of CS may increase the microbial membrane’s osmotic pressure-induced disruption and shrinkage [29]. Electrostatic interactions of CS can enhance the binding ability of its conjugated cationic AMP with the membrane components of both gram-positive and gram-negative bacteria and fungi [30,31]. In the FE-SEM analysis, significantly higher morphological alterations with surface disruptions, and in the PI uptake assay, higher levels of cell membrane permeability alterations were observed in both *C. albicans* and *A. baumannii* in the Octominin-CNPs-treated groups than in the Octominin groups. This can be defined as the synergistic effect of CS and Octominin on membrane disruption. Other studies have proposed that CS may form a barrier on the bacterial/fungal surface, leading to the depletion of absorption of nutrients into the cells [32]. Lack of nutrients can induce metabolic stress and ultimately lead to the ROS-mediated self-destruction of fungi and bacteria [33,34]. In this study, further enhanced permeabilization led to the internalization of high levels of Octominin and CS to induce metabolic stress, synergistically inducing ROS generation in *C. albicans* and *A. baumannii* and eventually causing fungal/bacterial cell death.

The positive charge of CS is predicted to react with electrostatically negatively charged microbes that settle on the surfaces to inhibit biofilm formation and CS can bind with biofilm components, such as extracellular polymeric substances, proteins, and DNA, resulting in the eradication activity of preformed biofilms [35,36]. The polymeric nature of CS allows chelation with metals, such as calcium, zinc, and magnesium, which are required for the transcription and translation of microbes, and also for the synthesis of metabolites for biofilm formation; thus, these processes become distressed, leading to cell death and biofilm formation inhibition [30,37]. The sustained release of Octominin from the Octominin-CNPs was an additional benefit for prolonged biofilm inhibition. Therefore, although Octominin was previously identified as a potent antibiofilm agent, encapsulation into CNPs induced its antibiofilm activity to a superior level by biofilm inhibition and eradication in both *C. albicans* and *A. baumannii*.

In conclusion, our data emphasize that Octominin encapsulation into CNPs is an efficient Trojan horse strategy to deliver Octominin not only into *C. albicans* and *A. baumannii*, but also into their biofilms. Furthermore, the biphasic release profile of the Octominin-CNPs was beneficial for rapid and sustained antimicrobial activity. The physicochemical properties of CS induced the antibacterial and antifungal activities of Octominin to the next degree in a synergistic manner. Octominin-CNPs produced higher antimicrobial activity in time kill-kinetics assay, morphological alterations, membrane permeability, ROS generation, and antibiofilm activity in both *C. albicans* and *A. baumannii* than in their similar concentrations of Octominin. This novel strategy might open a new destination for Octominin to develop as a final dosage form to overcome inherent limitations in therapeutic usage to combat MDR pathogens.

## 4. Materials and Methods

### 4.1. Optimization of Octominin Encapsulation into CNPs

Optimization of Octominin-CNPs encapsulation reaction was conducted using different ratios of Octominin (Table 1) by the ionotropic gelation method as described by Piras et al. [17]. Briefly, CS (Sigma-Aldrich, St. Louis, MO, USA) solution (1 mg/mL) was prepared by dissolving CS in 1% (*v*/*v*) acetic acid (pH 5), and CMC (Santa Cruz Biotechnology, Dallas, TX, USA) solution (1 mg/mL) was prepared by dissolving CMC in distilled water. CS and CMC solutions were filtered using a 0.1 μm Minisart^®^ syringe filter (Sartorius, Goettingen, Germany). Octominin was dissolved in nuclease-free water (1 mg/mL). For encapsulation, different volumes of Octominin were mixed with CMC (2 mL). Distilled water was added to equalize the volume of each reaction mixture. The mixture was then placed on a magnetic stirrer for 40 min for continuous mixing. Then, 0.4 mL of the CS solution was added to each mixture dropwise with continuous stirring. The final mixture was stirred for another 1.5 h. Encapsulated NPs were collected by centrifugation at 12,000 rpm for 30 min at 4 °C. The Octominin-CNPs or CNPs were separated from the supernatant and suspended in 1× phosphate-buffered saline (PBS). The supernatant was collected to calculate EE and LC. The particle size of the synthesized Octominin-CNPs was analyzed using a Mastersizer 3000 laser diffraction particle size analyzer (Malvern Panalytical, Malvern, UK).

### 4.2. Determination of the EE and LC of the Octominin-CNPs

After the encapsulation followed by centrifugation, the supernatant was used to measure the remaining peptide concentrations using a Nanodrop (Thermo Fisher, Waltham, MA, USA) to calculate the amount of encapsulated Octominin. EE and LC were calculated using the following equations:

$$\mathrm{EE}\;=\frac{\left(\mathrm{Initial}\;\mathrm{weight}\;\mathrm{of}\;\mathrm{Octominin}\;-\;\mathrm{Remained}\;\mathrm{weight}\;\mathrm{of}\;\mathrm{Octominin}\;\mathrm{in}\;\mathrm{supernatant}\right)}{\mathrm{Initial}\;\mathrm{weight}\;\mathrm{of}\;\mathrm{Octominin}}\times 100\%$$

$$\mathrm{LC}\;=\frac{\left(\mathrm{Initial}\;\mathrm{weight}\;\mathrm{of}\;\mathrm{Octominin}\;-\;\mathrm{Remained}\;\mathrm{weight}\;\mathrm{of}\;\mathrm{Octominin}\;\mathrm{in}\;\mathrm{supernatant}\right)}{\mathrm{Total}\;\mathrm{amount}\;\mathrm{of}\;\mathrm{CS}\;\mathrm{and}\;\mathrm{CMC}\;\mathrm{used}\;\mathrm{for}\;\mathrm{encapsulation}}\times 100\%$$

### 4.3. Determination of Release Kinetics Profile of the Octominin-CNPs

The highest EE and the best particle size generating reaction ratio (CS:CMC:Octominin-0.4:2:1) was selected for further encapsulation and experiments. To determine the release kinetics, 1 mg of Octominin-containing Octominin-CNPs was dissolved in 1 mL of PBS with mild sonication. The dissolved Octominin-CNPs mixture was placed on a rocker at 18 rpm for 24 h at room temperature (25 ± 2 °C). The mixture was centrifuged at 12,000 rpm for 30 min at 4 °C, the supernatant was removed, and the concentration of Octominin was measured using a NanoDrop One (Thermo Scientific, Waltham, MA, USA). The collected Octominin-CNPs were dissolved again in PBS (1 mL) with mild sonication and placed on a rocker under the above conditions. Peptide (Octominin) concentration was measured at every 24 h intervals to calculate the amount of released Octominin at each time point.

### 4.4. TEM Analysis of Octominin-CNPs

Morphological characterization of the Octominin-CNPs or CNPs was performed by TEM analysis. Briefly, samples were dissolved in PBS and 10 µL of the sample was placed on formvar/carbon-coated copper grid and incubated for 10 min. The excess sample amount was removed using filter paper. Then, 5 µL of 2% uranyl acetate (Sigma-Aldrich, St. Louis, MO, USA) was placed on the grid for 5 s, and the excess amount of uranyl acetate was removed by aspiration using filter paper. The dried grid was observed using a 300 keV Field emission–TEM (TecnaiTM G2 F30 super-twin (FEI), Hillsboro, OR, USA).

### 4.5. Determination of Cytotoxicity of Octominin-CNPs

To determine the cytotoxicity, cell viability was quantified by MTT assay using HEK 293 cells treated with Octominin-CNPs, Octominin, and CNPs. Cells were cultured in Dulbecco’s modified Eagle’s medium (Welgene, Gyeongsan-si, Gyeongsanbuk-do, Korea) containing 10% (*v*/*v*) fetal bovine serum (Welgene, Gyeongsan-si, Gyeongsanbuk-do, Korea) and an antibiotic-antimycotic solution (Welgene, Gyeongsan-si, Gyeongsanbuk-do, Korea) and incubated for 24 h in a humidified atmosphere of 5% CO_2_ at 37 °C. Cells were collected and seeded in 96 microplates at a density of 2.0 × 10^5^ cells/mL (100 µL/well) and allowed to adhere to the wells for 12 h. The culture media was replaced and treated with different concentrations of Octominin-CNPs and Octominin (0–400 µg/mL). After 24 h of incubation, the culture medium was replaced with fresh media (90 µL) and 10 µL of 5 µg/mL MTT (Sigma-Aldrich, St. Louis, MO, USA) and was incubated for 4 h at 37 °C. Then, 50 µL of dimethyl sulfoxide (DMSO; Sigma-Aldrich, St. Louis, MO, USA) was added after removing the culture media to solubilize the formazan dye, and absorbance was measured at OD_595_ using a microplate spectrophotometer (Bio-Rad, Saint Louis, MO, USA).

### 4.6. Determination of In Vivo Toxicity of Octominin-CNPs

Zebrafish mating, embryo collection, and maintenance were conducted according to the method described by Edirisighe et al. [38]. In brief, 60 h post-fertilized larvae (n = 10) were exposed to different concentrations (0, 10, 25, 50, and 100 µg/mL) of either Octominin-CNPs, Octominin, or CNPs for 96 h. Larval mortality was observed at 12 h intervals of post-treatment, and growth abnormalities and behavioral changes were examined for 96 h. Four larvae from each treatment group (at 96 hpt) were subjected to measure the ROS levels by staining with H_2_DCFDA (Sigma-Aldrich, St. Louis, MO, USA) according to the method described by Edirisighe et al. [38]. Briefly, larvae were exposed to H_2_DCFDA (5 μg/mL) for 30 min, and the excess stain was removed by washing thrice with embryonic media. One group of larvae was exposed to H_2_O_2_ (5 mM) for 15 min before staining and used as a positive control (PC). ROS levels were observed as green fluorescence under a light microscope (Leica KL300 LED, Wetzlar, Germany) equipped with a fluorescence filter (Nightsea, Hatfield, PA, USA).

### 4.7. Determination of the Antifungal and Antibacterial Activity of Octominin-CNPs against C. albicans and A. baumannii

For the antifungal activity tests, *C. albicans* was cultured in potato dextrose broth/agar medium and incubated at 37 °C. For determining antibacterial activity, *A. baumannii* was cultured in tryptic soy broth/agar medium and incubated at 25 °C. For each microbial treatment, Octominin-CNPs concentrations were calculated based on the amount of encapsulated Octominin. The microdilution susceptibility test was conducted to determine time-kill kinetics analysis according to the guidelines of the Clinical and Laboratory Standards Institute (CLSI), M07-A. *C. albicans* and *A. baumannii* were seeded into 96-well microplates (190 µL/well) in triplicates at a density of 1 × 10^6^ CFU/mL. Ten microliters of Octominin-CNPs and Octominin were treated with different concentrations (0–300 µg/mL for *C. albicans* and 0–50 µg/mL for *A. baumannii*) and incubated for 24 h. Fungal/bacterial growth was measured at OD_595_ at each 3 h interval (0, 3, 6, 9, 12, 15, 18, 21, and 24 h) using a spectrophotometer (Bio-Rad, Saint Louis, MO, USA).

Changes in fungal and bacterial viability with Octominin-CNPs and Octominin treatments were quantified by MTT assay. Initially, *C. albicans* and *A. baumannii* were seeded on 96 microwell plates at a density of 1 × 10^6^ CFU/mL and treated with Octominin-CNPs, Octominin, and CNPs (100 and 200 µg/mL for *C. albicans*, and 5 and 10 µg/mL for *A. baumannii*). Each microorganism was treated with PBS as a negative control, fluconazole as a PC for *C. albicans*, and chloramphenicol as a PC for *A. baumannii*. The *C. albicans* and *A. baumannii* plates were incubated for 24 h at 37 °C and 25 °C, respectively. Cells were collected by centrifugation at 1500× *g* for 10 min and washed with PBS. The MTT assay was conducted according to the method described above for the cytotoxicity assay, and absorbance was measured at OD_595_ using a microplate spectrophotometer.

### 4.8. Determination of Morphological Changes of C. albicans and A. baumannii with Octominin-CNPs Treatment

FE-SEM analysis was conducted on Octominin-CNPs, Octominin, and CNPstreated *C. albicans* and *A. baumannii* to determine ultrastructural changes. Fungi and bacteria were cultured as described in Section 4.7, and a cell density of 1 × 10^6^ CFU/mL was used for the treatments. Octominin-CNPs and Octominin were treated at 50 and 200 µg/mL for *C. albicans* and 5 and 10 µg/mL for *A. baumannii* and incubated for 9 h at 37 °C and 25 °C, respectively. Each microorganism was treated with the CNPs and PBS as the negative control groups. Cells were collected by centrifugation at 1500× *g* for 10 min, washed with PBS, and pre-fixed with glutaraldehyde (5%) for 20 min. After washing with PBS, samples were dehydrated with serial dilutions of ethanol (30, 50, 70, 80, 90, and 100%). The platinum coating was performed via ion sputtering (E-1030, Hitachi, Tokyo, Japan), and the samples were observed using FE-SEM (Sirion FEI, Eindhoven, The Netherlands).

### 4.9. Determination of Membrane Permeability Alteration and ROS Generation of C. albicans and A. baumannii with Octominin-CNPs Treatment

PI coupled with FDA and H_2_DCFDA staining assays were conducted on Octominin-CNPs, Octominin, and CNPs treated *C. albicans* and *A. baumannii* to determine the efficiency of cell membrane permeability alterations and ROS generation, respectively. Candida and bacterial cultures were prepared at a cell density of 1 × 10^6^ CFU/mL according to the aforementioned conditions. Two milliliters of broth were treated with Octominin-CNPs, Octominin, and CNPs at 50 and 200 µg/mL for *C. albicans* and 5 and 10 µg/mL for *A. baumannii*. Negative control (PBS) treatment was conducted, and cultures were incubated for 12 h according to the aforementioned conditions. *C. albicans* and *A. baumannii* were isolated by centrifugation at 1500× *g* for 10 min. Isolated cell pellets were washed with PBS and resuspended in PBS. For permeability alteration monitoring, 1 mL of each suspension was stained with 50 μg/mL of PI (Sigma Aldrich, Saint Louis, MO, USA) and 40 μg/mL of FDA (Sigma Aldrich, Saint Louis, MO, USA), incubated for 30 min in the dark. To determine ROS generation, 1 mL of each cell suspension was stained with 50 μg/mL H_2_DCFDA and incubated for 30 min in the dark. Excess dye was removed by centrifugation at 1500× *g* and the cells were washed with PBS three times. The cell pellet was resuspended in 20 µL of PBS, and 5 µL of the suspension was placed on a glass slide and observed under a confocal laser scanning microscope (Carl Zeiss, Jena, Germany). In the PI uptake assay, red fluorescence was observed to determine dead and membrane permeability-altered cells, and live cells were observed by green fluorescence. To determine ROS generation, green fluorescence was monitored in H_2_DCFDA-stained samples. The excitation and emission wavelengths for red fluorescence were 535 and 617 nm, respectively, and those for green fluorescence were 488 and 535 nm, respectively.

### 4.10. Determination of C. albicans and A. baumannii Biofilm Inhibition and Eradication Activity of Octominin-CNPs

A CV-staining-based biofilm quantification method was used to determine the biofilm inhibition and eradication activities in Octominin-CNPs treated *C. albicans* and *A. baumannii*. For the biofilm inhibition assay, Candida and bacterial broth at 1 × 10^6^ CFU/mL were placed in 96-microwell plates (200 µL/well) and treated with Octominin-CNPs, Octominin, and CNPs at concentrations of 50 and 200 µg/mL for *C. albicans* and 5 and 10 µg/mL for *A. baumannii*. The PBS-treated group was included as a control group. For the biofilm eradication assay, initially, *C. albicans* and *A. baumannii* were seeded at a density of 1 × 10^6^ CFU/mL and placed in a 96-microwell plate (200 µL/well) and allowed to form biofilms for 24 h incubation. The supernatant was removed, the biofilm was carefully washed with PBS, replaced with the media in each well, and treated with Octominin-CNPs, Octominin, and CNPs at 50 and 200 µg/mL for *C. albicans* and 5 and 10 µg/mL for *A. baumannii*. PBS (negative control) treatment was also conducted, and the plates were incubated for 24 h. CV staining was conducted on the biofilm inhibition and biofilm eradication assay plates to quantify the remaining biofilm after each treatment. Initially, the remaining supernatant was carefully removed, and the biofilms were washed with PBS. Samples were fixed for 10 min with 100% methanol. After removing methanol, the biofilm was stained with 0.1% (*w*/*v*) of CV (Sigma-Aldrich, St. Louis, MO, USA) for 30 min at room temperature (26 ± 2 °C). Excess CV was removed, and the biofilm was washed thrice with PBS. Finally, the CV-stained biofilm was dissolved in 95% ethanol and agitated. The absorbance of the supernatant was measured at OD_595_ using a microplate spectrophotometer. The biofilm inhibition/eradication percentages were quantified using the same formula. Biofilm formation inhibition/eradication% = [1 − (Ab test/Ab negative control)] × 100, where the Ab test represents the absorbance value of Octominin-CNPs or Octominin, and Ab negative control represents the absorbance of the negative control (PBS).

### 4.11. Statistical Analysis

All experimental data were analyzed using GraphPad Prism version 8 (GraphPad Software Inc., La Jolla, CA, USA). One-way analysis of variance (ANOVA) and/or unpaired *t*-tests were performed to determine statistically significant (*p* < 0.05) differences between the control and the treatments of MTT, biofilm inhibition, and biofilm eradication assays. The in vivo survival data were analyzed using Log-rank (Mantel–Cox) test data and are shown as the mean ± standard deviation (SD) of triplicate experiments.

## Figures and Tables

**Table 1:** Characterization of Octominin-CNPs derived from different CS:CMC:Octominin reaction mixtures.

	Reaction 1	Reaction 2	Reaction 3	Reaction 4	Reaction 5
CS:CMC:Octominin	0.4:2:0	0.4:2:0.25	0.4:2:0.5	0.4:2: 1	0.4:2:1.5
Particle diameter (nm)	246.81 ± 1.98	552.86 ± 19.24	364.57 ± 11.78	372.80 ± 2.3	237.60 ± 14.37
PDI	0.24 ± 0.014	0.86 ± 0.188	0.22 ± 0.008	0.24 ± 0.009	0.41 ± 0.077
EE (%)	NA	94.8	94.8	96.49	78.16
LC (%)	NA	9.88	19.75	40.20	48.85

Polydispersity index—PDI, encapsulation efficiency—EE, and loading capacity—LC.
